# A Revised Mixed-Approach Rubric for the Quality of Academic Posters

**DOI:** 10.3390/pharmacy13050134

**Published:** 2025-09-17

**Authors:** Michael J. Peeters, Megan A. Kaun, Kimberly A. Schmude

**Affiliations:** College of Pharmacy & Pharmaceutical Sciences, University of Toledo, Toledo, OH 43614, USA; megan.kaun@utoledo.edu (M.A.K.); kimberly.schmude@utoledo.edu (K.A.S.)

**Keywords:** rubric, poster quality, validity, validation

## Abstract

The quality of posters at pharmacy conferences can vary. We created a mixed-approach rubric (MAR) for poster quality. Evidence from multiple sources (systematic review, further analysis of rater scores, verbal feedback from raters) showed the need to slightly modify that MAR, which we accomplished. Our objectives here were to re-evaluate scoring using this revised MAR (rMAR) and to further examine the attributes of lower-quality versus higher-quality posters. Two faculty raters independently scored each poster using the rMAR for recent posters presented at a pharmacy education conference. The Rasch Measurement Model provided psychometric evidence and poster-quality measures. These measures were then linear-regressed with attributes of logical sequencing, QR-code presence/use, submission abstract presence, and wordiness. Moreover, Traditional vs. Contemporary poster formats were compared. Raters scored 642 posters (267 from 2023, 375 from 2024). The Rasch Measurement Model showed a distinct separation of posters into lower quality versus higher quality. The rMAR’s rating scale continued to function well (like the original MAR had) among multiple raters. Poster-quality measures were significantly positive when linearly regressed with logical sequencing, QR-code presence/use, absence of submission abstract, and decreased wordiness. Moreover, Contemporary poster formats (either Persky-style or Billboard-style) were higher quality on average than Traditional poster formats. This evidence-based rMAR showed a helpful validation of poster-quality scores. Regression confirmed findings from the initial MAR (before revision), and choice of poster format proved a notable decision affecting poster quality.

## 1. Introduction

Poster presentations are a common activity at pharmacy conferences and a means to both disseminate research findings and facilitate professional development [1,2]. However, they can differ in quality, with consequential results on communicating poster content [1,2,3,4,5]. Thus, poster quality appears important for quantification. And even dichotomous quantification should help facilitate discovery of helpful versus hurtful poster characteristics that affect poster quality and communication among the community of scholars.

Rubrics are a notable tool for this purpose of quantifying, though there are multiple types of rubrics. The two most common types of rubrics are analytic rubrics and holistic rubrics [6]. Analytic rubrics are what many pharmacy educators describe as simply a “rubric” or a “standardized rubric” [7] and have scoring on each of multiple criteria that are summed into a total score [6,7], whereas lesser-known holistic rubrics are the opposite and have one total score criterion to evaluate for the entirety (or whole) of the artifact [6]. A systematic review of poster rubrics in 2023 had a few notable takeaways. First, it revealed that the majority of the 12 identified poster-quality rubrics were analytic (n = 9) while a minority were holistic (n = 3) [8]. Second, there was very little psychometric evidence for scores from any of the rubrics found. Of those with psychometric evidence, the evidence for rigor in creating those rubrics was mostly limited only to reporting a sample-dependent reliability coefficient. Third, no rubric had been created using Modern Test Theory such as Item Response Theory or Rasch Measurement. (While all prior rubrics had used Classical Test Theory, the rigorous Rasch Measurement Model used herein is notable for its validity evidence [9,10] and its sample-*in*dependence/invariance [11] of scores).

While these are the most common types of rubrics, a third type—mixed-approach rubrics—is an integration of the two common, disparate analytic and holistic rubric types [12]. The mixed-approach rubric is a less-common type of rubric that has attempted to merge the advantages and avoid the disadvantages of the analytic and holistic rubric types. With no mixed-approach rubric identified in the literature [8], we subsequently created a mixed-approach rubric for poster quality (MAR) [2]. It included common categories from analytic rubrics in the literature, though it used the quick single-rating-scale scoring of a holistic rubric to measure poster quality [12]. In the year after creating it, we empirically investigated this MAR in comparison to other rubrics, where it showed favorable results to both analytic [13] and holistic [14] rubrics. The MAR proved quicker to score while producing similar results to one analytic rubric and *better* than (more reliable) scores from another analytic rubric [13]. Meanwhile, when the mixed-approach scoring with the MAR was compared with holistic scoring, scoring with the MAR was quite similar in time to score though more accurate when comparing less-experienced raters with more-experienced raters; scoring by using the MAR appeared to help focus and guide less-experienced raters towards making the same decisions as more-experienced raters [14]. Of note, both these studies [13,14], in general, were confirmatory validation evidence for scoring with this MAR, as both studies had been performed using posters from a subsequent year (2023) of the same annual meeting from the original MAR [2] creation that used 2022 posters (i.e., different posters).

**Figure 1:**
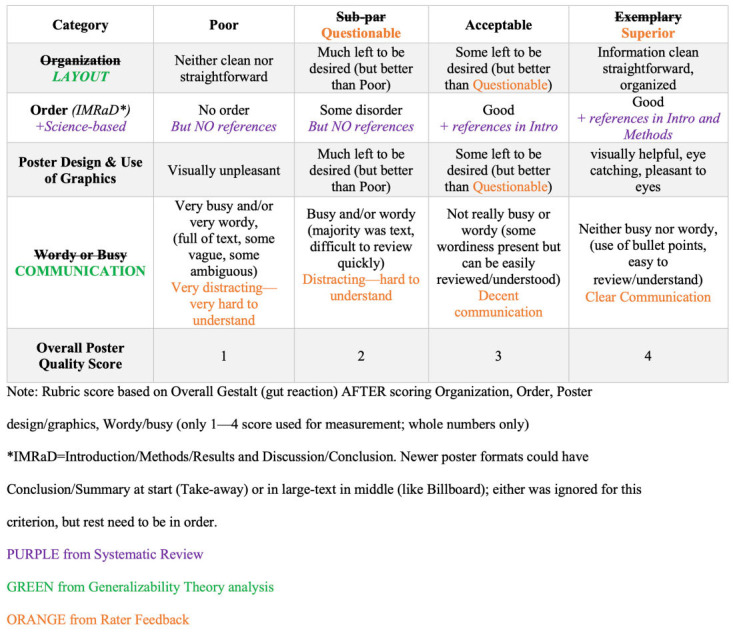
Original mixed-approach rubric for poster quality (MAR), with superimposed changes from subsequent evidence.

Various sources of evidence surfaced following our initial MAR creation. First, when we more closely scrutinized and compared the original MAR to other non-rubric findings in the 2023 systematic review [8], we discovered that the original MAR was missing “scientific rigor”. Second, a multivariate Generalizability Study (In brief, the multivariate Generalizability Theory followed the multivariate analysis approach described by Brennan [15] and used *lme4* package version 1.1-9 within R [fully crossed design of poster × rater:category (p × r°:c^∙^)] of raters’ mixed-approach scores for the rubric’s four categories of: (a) Organization, (b) Order, (c) Poster design and use of graphics, and (d) Wordy or busy.) of sub-scores from the holistic study [14] that were not analyzed or published within that report found that scores for the “Order” and “Organization” categories correlated with one another at 0.80 (high), while scores from the “Design” and “Wordy” categories correlated at 0.84 (high) with one another; all other categories were less than 0.65 (lower). Both high correlations suggested a large degree of overlap and potential redundancy when compared with other lower correlations. This should be addressed in an attempt to make all rubric categories distinct from each other and so allow the best statistical discrimination among rubric categories. Third, we used feedback provided from raters in the various studies [13,14] using the MAR. Figure 1 shows the initial MAR with potential changes noted from these empirical evidence sources.

Given that we modified the original MAR, our objectives for this investigation were to re-evaluate (revalidate) scores from the use of this revised MAR (rMAR) and then to further characterize poster attributes that may enhance or diminish a poster’s quality.

## 2. Materials and Methods

**Figure 2:**
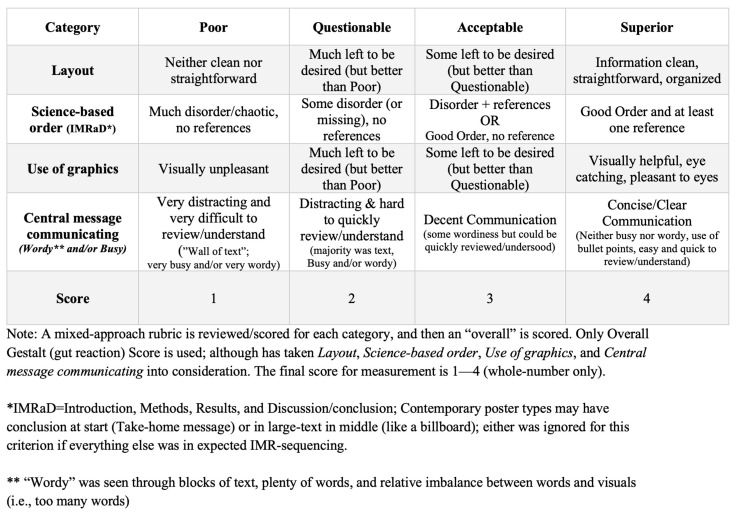
The revised mixed-approach rubric (rMAR) for evaluating quality of academic posters.

Based on evidence in the Introduction, we revised the MAR. Figure 2 shows this rMAR and can be compared to the original MAR in Figure 1 to see specific changes.

*Setting for rMAR use*: Two experienced faculty members independently scored each and every research poster from an electronic poster repository of the American Association of Colleges of Pharmacy (AACP) for Annual Meetings in 2023 (raterA and raterB) and 2024 (raterA and raterC), using this rMAR. Of note, the experienced faculty members have been involved with creating, viewing, and judging posters for decades. Notably, scoring with the rMAR did not require them to understand the intricacies of all poster contents/details; instead, the rMAR measures poster quality according to multidisciplinary criteria for what makes a good poster, including visuals/graphics use and limiting wordiness. After rMAR scoring, the data were modeled using the Many-Facets Rasch Measurement [16]. (The Rasch Measurement Model can provide sound validity [9,10] and validation [10,17] evidence.) Additionally, training of raters was minimal, with a one-hour-long meeting in which the raters were oriented to the rMAR and scored a few posters to discuss scoring.

**Figure 3:**
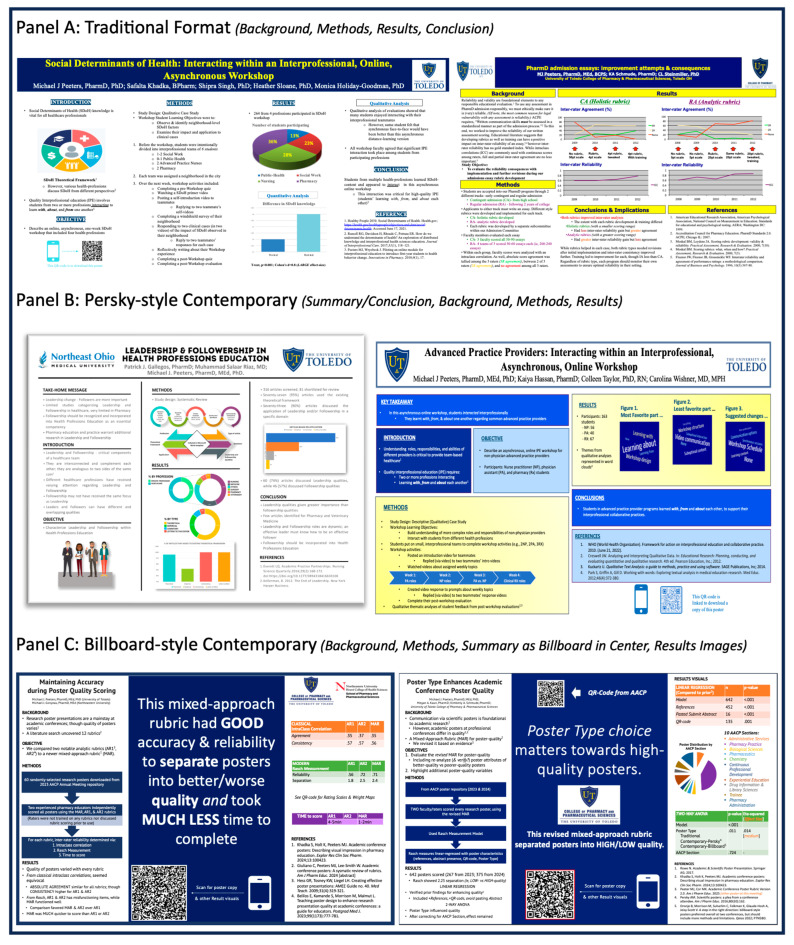
(**A**–**C**) Poster format examples. (Note for Reading: Focus on placement of section headers. Other details of these posters need not be read and may be too small for some readers to appreciate).

Along with rMAR scoring, the raters also coded for poster attributes of whether the submitted abstract was on the poster; whether it was logically sequenced (had Introduction/Methods/Results/Conclusion as academicians expect); whether it seemed wordy; whether it had/used QR-code(s); and whether the poster format was Traditional [18], Persky-style Contemporary [19], or Billboard-style Contemporary [20]. (Poster Format Note: Three poster formats were categorized. The most familiar format was labeled *Traditional*. Readers will undoubtedly be familiar with its conventional IMRaD ordering. Examples are shown in Figure 3A. The two others were variants of Contemporary, as both formats highlighted the poster’s summary and deviated from the Traditional format. We labeled the second format as *Persky-style Contemporary*, which provides the poster summary (often called “Take-away” within these posters) at the top left, and examples are shown in Figure 3B. The third was *Billboard-style Contemporary* and is so named because it follows with the idea of a car driving by a Billboard, in that its central message is front and center on the poster, so that passers-by can quickly ascertain the poster’s central message. Examples of this format are shown in Figure 3C. Notably, the main-text reference for each format can provide further examples.) 

*rMAR Rating Scale*: One important but often over-looked aspect of helpful psychometric measurement is how raters use the instrument’s rating scale [10,11,21,22,23]. Linacre has suggested Rasch Measurement Model criteria to evaluate the rating scale [22]; we used Linacre’s framework including having at least 10 observations per scale category, OUTFIT mean squares < 2.0, and rating-scale categories advancing sequentially (hopefully by at least 1.4 logits).

*Linear Regression to Characterize Poster Attributes*: Based on the raters’ scores with rMAR, the Rasch Measurement Model analyzed poster-quality measures [16,21]. Minifac version 3.83.1 (winsteps.com, accessed on 9 September 2025), a freely available version of Facets, was used for this investigation. We used the adjusted (or fair) measures in this analysis, as the Rasch Measurement Model can provide fair measures adjusted for severity of raters (as some raters might use the same rating scale more harshly or leniently than other raters [16]).

Those fair measures were imputed into a spreadsheet containing other poster attributes of whether the submitted abstract was on the poster, whether it was logically sequenced (had Introduction/Methods/Results/Conclusion as academicians expect), whether it seemed wordy, whether it had/used QR-code(s), and poster format (whether Traditional, Persky-style Contemporary, or Billboard-style Contemporary).

Using linear regression in SPSS version 29 (IBM), poster-quality measures were regressed with poster attributes of whether the submission abstract was pasted onto the poster (i.e., wordiness), whether it had references, and whether the poster used QR-code(s). (Note: These attributes were previously analyzed within the initial rubric investigation; we aimed to examine whether this evidence was confirming or not.)

*Further poster analysis*: Furthermore, and because the entire collection of posters was reviewed, we could examine the role of poster format (Traditional, Persky-style Contemporary, or Billboard-style Contemporary) and AACP Section (unit of professional organization that the primary poster author is foremost associated with). We first analyzed poster format with a 1-way ANOVA (which could allow Bonferroni-corrected comparisons among the three formats). Thereafter, we analyzed these together using a 2-way ANOVA, to determine whether either or both had an independent effect on the quality of posters. For the 1-way and 2-way ANOVAs, we moved beyond statistical significance alone to explain practical significance using partial eta-squared for ANOVA effect-size interpretations [24]. SPSS version 29 (IBM) was used for this as well.

## 3. Results

**Figure 4:**
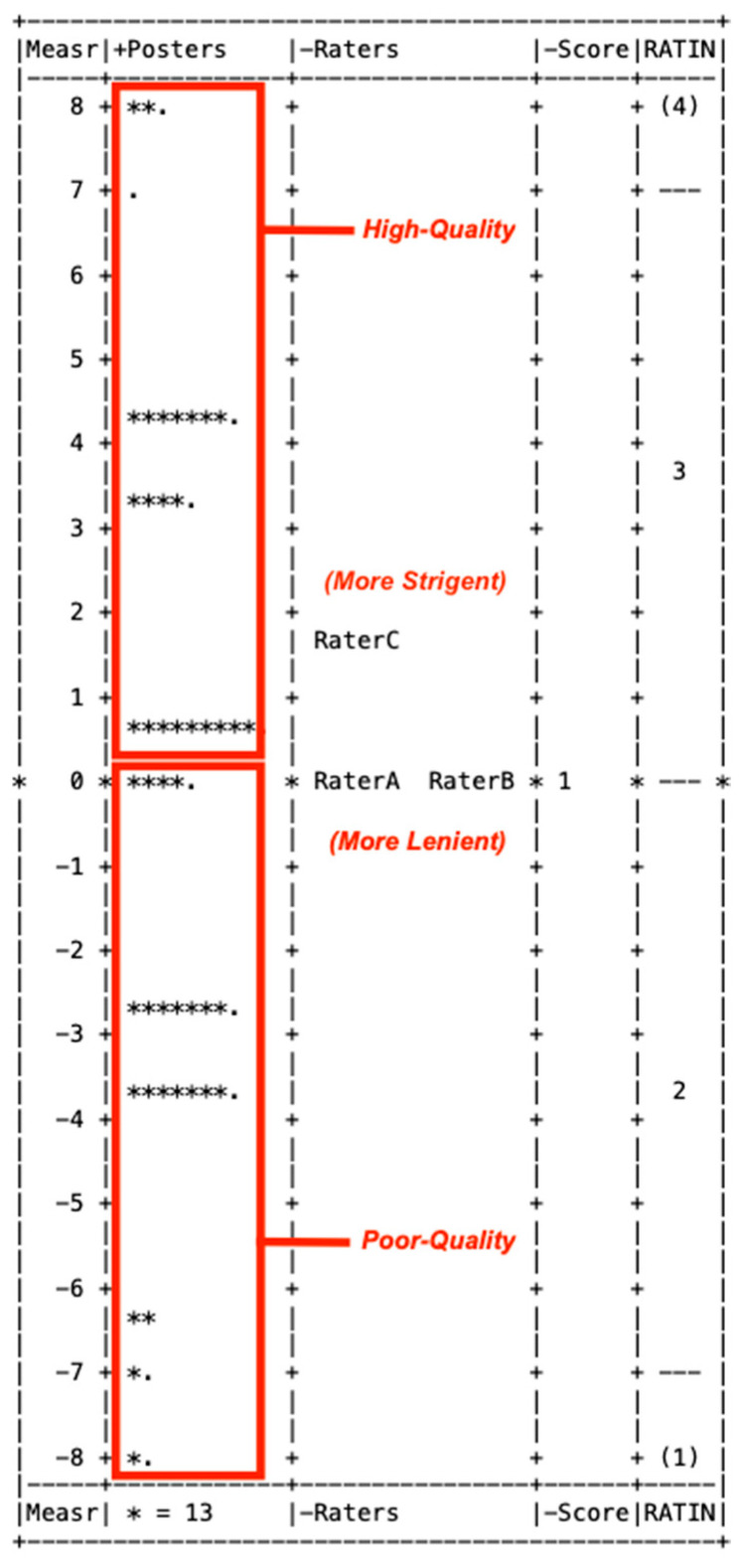
Wright Map for scoring of 642 academic conference posters with a revised mixed-approach rubric (rMAR).

*Evaluation of rMAR*: From the electronic repository, raters scored 642 posters (267 from 2023 and 375 from 2024). Importantly, the model separation was 2.25 (i.e., could divide into at least two groups of low versus high quality) with 0.67 reliability. Figure 4 is a Wright Map (person–item map) illustrating the distribution of posters based on the single-item rMAR and provides a good, single-picture summary as psychometric evidence for this rMAR. It showed a good distribution of poster-quality measures. The average poster-quality measure was −0.21 logits ±4.07. In terms of fit to the Rasch Measurement Model, the average infit and outfit were both a mean square of 0.78.

**Figure 5:**
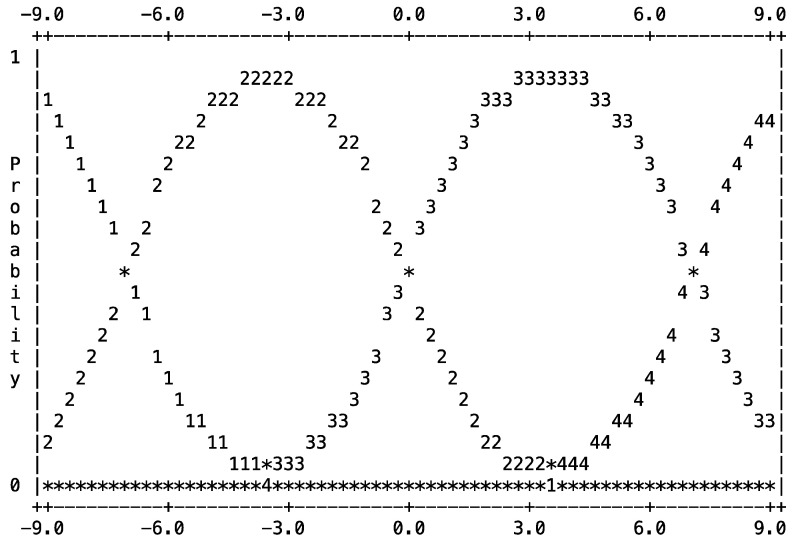
The rating scale for 1 item of a revised mixed-approach rubric (rMAR) that was used to score the quality of 642 posters, as used by three faculty raters. (Note for Reading: This rating scale probability curve ideally should look like a series of “hills”; each should have a distinct peak, and they should not overlap (which would suggest difficulty among raters in using that rating scale). The rating-scale numbers increased sequentially, and there was no overlap seen in the peaks. This rating scale appeared to function well.)

*rMAR Rating Scale*: While raters showed some variation (*p* < 0.001), their use of the rMAR’s four-point rating scale appeared helpful; this rating scale functioned well among these raters. Raters used all categories with 5% as 1, 50% as 2, 42% as 3, and 3% as 4. The OUTFIT of rating-scale categories was 0.9–1.0 (very good), while rating-scale thresholds were approximately 7 logits per category change. With rating-scale categories ordered sequentially and each showing a distinctive peak, Figure 5 shows the probability curve for the rubric’s single-item rating scale, as used by the multiple faculty raters.

*Linear Regression*: Poster-quality measures positively regressed with presence of logical sequencing (t = 6.7, *p* < 0.001), presence/use of QR-code (t = 2.2, *p* = 0.032), absence of submission abstract (t = 4.1, *p* < 0.001), and decreased wordiness (t = 11.7, *p* < 0.001). Moreover, contemporary poster formats were higher quality on average than the Traditional poster format (t = 3.0, *p* = 0.003).

*Poster Format*: Furthermore, the Traditional poster format was 83% (535/642), while the Persky-style Contemporary poster format was 7% (42/642), and the Billboard-style Contemporary poster format was 10% (65/642). From one-way ANOVA, choosing a Traditional poster format led to lower quality than using either Persky-style or Billboard-style Contemporary poster formats (*p* < 0.001; partial eta-squared = 0.03, medium effect size). Bonferroni-adjusted post hoc comparisons showed that Persky-style Contemporary was higher quality than Traditional (*p* = 0.005); Billboard-style Contemporary was higher quality than Traditional (*p* = 0.003), and Persky-style Contemporary did not appear different from Billboard-style Contemporary poster format (*p* > 0.99).

As shown in Table 1, poster format had variations among the AACP Sections. While one-way ANOVA analysis suggested that poster quality varied by AACP Section, a more extensive two-way ANOVA adjusted for poster format and showed it to be secondary to differences from poster format [*p* < 0.001 overall; partial eta-squared = 0.02, medium effect size; *p* = 0.01 for poster format; *p* = 0.159 for AACP Section].

## 4. Discussion

We met our objectives within this investigation; we revalidated scores from the rMAR and identified poster attributes that influenced quality. Our use of a Rasch Measurement Model produced rigorous evidence for revalidation of scoring. Moreover, a combination of a large sample size of posters, scoring posters from multiple years (from different annual meetings than the initial MAR creation data), and using the Rasch Measurement Model to characterize reliability and internal structure also provided sound evidence towards inference of generalization.

*rMAR*: Notably, our revisions to the original MAR [2] were based on multiple (three) sources of evidence. The first evidence was a systematic literature review of poster guidance articles [8]. As that systematic review was not limited to pharmacy and included many other disciplines, it would suggest that this rMAR should have promise for use beyond pharmacy. The second evidence was multivariate Generalizability Theory analysis of MAR sub-scores from a prior study [14]. The third evidence was from rater feedback, as raters suggested slight changes that could make their use of the MAR even better.

*rMAR Revalidation*: Sound measurement requires stringent validation evidence. Any modification to a rubric should be re-validated [25], and this was achieved in this instance. The rMAR’s fit to the Rasch Measurement Model becomes stringent evidence to support this rMAR as a rigorous poster-quality scoring tool. The separation into slightly more than two groups confirmed this, as the rMAR was able to differentiate poster quality along a continuum and distinctly separated posters into high versus low quality (as seen in Figure 4), and the four-point rating scale functioned well also (Figure 5). These findings, along with having used the underlying sample size of posters from multiple and subsequent years, served to revalidate scoring from this rMAR, establishing it as a sound evaluation tool (Figure 2).

*Characteristics Influencing Poster Quality*: This investigation confirmed previous findings that logical sequencing, presence of QR-code, exclusion of submission abstract, and limited wordiness were positive influences on poster quality [2]. This is further validation evidence for inferences of scoring and generalization. The presence of QR-codes, which provided supplemental content such as chemical diagrams, equations, or further pictures beyond the central image, did have a small but positive impact on poster-quality measures. QR-codes can be helpful to “de-clutter” and offload numerous images or figures that authors would like to include but that are not needed for the posters’ central message. These support prior recommendations that certain poster design choices can serve to enhance a poster’s quality and effectiveness [8].

In addition to the characteristics listed above, poster format demonstrated a notable role in the quality of a poster. While this was previously noted with dichotomous poster formats (Traditional compared with any format of Contemporary) [2], we further discriminated into three poster formats described in the literature (Traditional, Persky-style Contemporary, and Billboard-style Contemporary). Of note, we observed and scored some Traditional-format posters as superior (score = 4), while other Contemporary-format posters were sub-par (score = 2); although, on average, Contemporary formats did appear to be higher quality than the Traditional format, and the two Contemporary formats, Persky vs. Billboard, did appear similar on quality measure. Moreover, the effect size for poster format was *moderate*, which demonstrates that the initial poster-format decision appears to have a substantive impact on the quality of poster communication. Additionally, the AACP Section appeared to have an influence on poster quality if analyzed alone; however, this effect with the AACP Section appeared secondary to poster-format choice. In other words, it appears that some AACP sections may experiment more (or sooner) with advancement in poster format and therefore communicate better than others through their higher-quality posters.

*Contributions and Limitations*: This study applied the advanced psychometric Rasch Measurement Model to evaluate the quality of academic posters. The Rasch Measurement Model provides more-rigorous validation evidence than the Classical Test Theory approach that was initially used, especially across raters.

Despite these contributions, opportunities for continued enhancement of this rMAR exist. A foremost limitation is that, while a large sample size of posters was used that included multiple annual meetings, generalizability of the rMAR to settings outside of pharmacy education and the pharmacy profession can be further evaluated. A further limitation can be seen in Table 1, as some AACP sections did not yet have sample sizes of posters large enough to be definitively confident in exact statistical comparisons (>30 is often suggested for statistical testing [26]); however, the trends observed seem plausible in the investigators’ experiences (and the experiences of other colleagues). Additionally, poster guidance can differ among meetings and so a poster author may have experience with poster guidance from other meetings that a researcher frequents and that meeting’s poster norms may differ from this convention.

*Implications*: Posters are a common intermediate step to disseminate current research findings, enable professional development of pharmacy researchers regardless of their research focus (from pharmacy practice researchers to health service researchers to biological science researchers to medicinal chemistry researchers), and facilitate networking by similarly interested conference attendees. Regardless of purpose, high-quality posters are foundational for effective communication. Following from this revalidation of scoring from this rMAR, the psychometric evidence for this rMAR far surpasses any previous poster-quality rubrics. It seems prudent that this rMAR, with its notable and multiple psychometric evidence for its scores along with this report [2,13,14], should be used to score poster quality for conference poster competitions. Further and more importantly, this rMAR can be used by authors as they develop their posters. That is, along with a poster draft, a poster author could provide this rMAR (Figure 2) to their research mentor (or other colleague not involved in the poster development), for that mentor/colleague to circle/score each rMAR criterion and facilitate feedback of what has been shown to characterize high-quality posters across disciplines. Over multiple drafts, posters should evolve towards higher quality. As many posters within this investigation were scored as “sub-par”, there seems to be room for improvement; using the rMAR may help a mentor/colleague to identify lower-scoring criteria that could enhance overall poster quality, if addressed.

## 5. Conclusions

For measuring poster quality, scoring from the rMAR appeared to perform similar to scoring from the initial MAR, using the Rasch Measurement’s psychometric rigor. Having evaluated many more posters from subsequent years using this rMAR, differences related to different poster formats were a notable further finding. A poster author’s choice of a Contemporary poster format appeared to elicit higher quality measures on average than the choice of a Traditional poster format. This rMAR should be used to judge pharmacy conference posters and for the development of high-quality posters that communicate well.

## Figures and Tables

**Table 1:** Poster formats chosen by authors, and poster author affiliation within a professional organization (AACP).

AACP Section	Posters (n = 642)	Mean Quality Measure (SD) *	Traditional Poster Format [19]	Persky-Style Contemporary Poster Format [20]	Billboard-Style Contemporary Poster Format [21]
Administrative Services	50	2.6 (0.5)	40 (80%)	7 (14%)	3 (6%)
Pharmacy Practice	266	2.4 (0.6)	211 (79%)	18 (7%)	37 (14%)
Biological Sciences	26	2.1 (0.5)	24 (92%)	2 (8%)	0 (0%)
Pharmaceutics	25	2.1 (0.7)	25 (100%)	0 (0%)	0 (0%)
Chemistry	16	2.0 (0.7)	15 (94%)	0 (0%)	1 (6%)
Continuous Professional Development	26	2.5 (0.6)	17 (65%)	3 (12%)	6 (23%)
Experiential Education	72	2.4 (0.5)	65 (90%)	2 (3%)	5 (7%)
Drug Information and Library Sciences	24	2.6 (0.5)	19 (79%)	2 (8%)	3 (13%)
Trainees	47	2.5 (0.5)	45 (96%)	0 (0%)	2 (4%)
Pharmacy Administrative Sciences	90	2.3 (0.7)	74 (82%)	8 (9%)	8 (9%)
Overall	642	2.4 (0.6)	535 (83%)	42 (7%)	65 (10%)

* Poster-quality measure in 1–4 range and from score of revised mixed-approach rubric (rMAR).

## Data Availability

The raw data supporting the conclusions of this article will be made available by the authors on request.
